# The efficacy and safety of acupuncture assisted anesthesia (AAA) for postoperative pain of thoracoscopy: A protocol for systematic review and meta-analysis of randomized controlled trials

**DOI:** 10.1097/MD.0000000000028675

**Published:** 2022-01-28

**Authors:** Xinyi Liu, Dan Meng, Qinyu Zhao, Chunchun Yan, Jingyu Wang

**Affiliations:** a Institute of Literature and Culture of Traditional Chinese Medicine, Shandong University of Traditional Chinese Medicine, Jinan, Shandong, China; b Institute of Innovation Research of traditional Chinese Medicine, Shandong University of Traditional Chinese Medicine, Jinan, Shandong, China; c Institute of Acupuncture, Moxibustion, and Massage, Shandong University of Traditional Chinese Medicine, Jinan, Shandong, China.

**Keywords:** acupuncture assisted anesthesia, postoperative pain, protocol, systematic review and meta-analysis, thoracoscopy

## Abstract

**Background:**

Enhanced recovery after surgery suggests the use of multimodal analgesia to optimize the perioperative pain management scheme. At present, studies have shown that the application of acupuncture combined anesthesia in thoracoscopy has achieved good curative effect. However, there is no relevant systematic evaluation. Our study is the first meta-analysis of the effectiveness and safety of acupuncture combined anesthesia in pain management after thoracoscopy, in order to provide strong evidence for clinical support.

**Methods:**

A comprehensive and systematic literature searching will mainly perform on 7 electronic databases (PubMed, the Cochrane Library, Embase, China National Knowledge Infrastructure, Chongqing VIP Information, and WanFang Data, Chinese Biomedical Database) from their inception up to November 30, 2021. We will also search for ongoing or unpublished studies from other websites (eg, PROSPERO, ClinicalTrials.gov, Chinese Clinical Trial Registry) and do manual retrieval for potential gray literature. Only the relevant randomized controlled trials published in English or Chinese were included. Two independent investigators will independently complete literature selection, assessment of risk bias, and data extraction, the disagreements will be discussed with the third party for final decisions. The primary outcome measures: visual analog scale, intraoperative anesthetic dosage, and the consumption of postoperative analgesics. The secondary outcome measures: Pittsburgh Sleep Quality Index, the total sleep time after operation, residence time in the anesthesia recovery room, the duration of hospitalization, and the incidence of adverse reactions and serious events. Assessment of bias risk will follow the Cochrane risk of bias tool. Data processing will be conducted by Stata 15.0 software.

**Results:**

We will evaluate the efficacy and safety of acupuncture assisted anesthesia for postoperative pain after thoracoscopy based on randomized controlled trials.

**Conclusion:**

This study can provide more comprehensive and strong evidence whether acupuncture assisted anesthesia is efficacy and safe for postoperative pain in thoracoscopy.

**Registration:**

The research has been registered and approved on the INPLASY website. The registration number is INPLASY 2021120129.

## 1 Introduction

Compared with traditional thoracotomy, thoracoscopy has certain advantages in reducing surgical trauma, acute and chronic pain, and postoperative complications.^[1]^ However, in all surgical operations, even today when video assisted thoracic surgery is widely used in thoracic surgery, the pain after thoracoscopy is still in the forefront of all surgical treatments.^[2]^ The pain of thoracoscopy can be divided into the pain of the wound itself, the pain caused by the traction of the drainage tube, and the active pain caused by routine atomization inhalation, back buckle, deep breathing, and effective cough to prevent pulmonary infection.^[3]^ Inadequate analgesia after thoracic surgery may lead to stress response, pulmonary infection and other complications, anxiety, depression, insomnia, and other reactions, which may affect the postoperative recovery. Therefore, effective control of pain after thoracoscopy has been paid more and more attention. Lung surgery guidelines strongly recommend the retention of opioids for analgesia.^[4]^ However, the use of opioids will produce more side effects and adverse reactions, and there is a certain correlation between the dosage and adverse reactions.^[5]^ Therefore, enhanced recovery after surgery (ERAS) suggests the use of multimodal analgesia to optimize the perioperative pain management scheme.^[6–7]^

At present, multimodal analgesia after thoracic surgery mainly includes general anesthesia combined with paravertebral nerve block, patient-controlled epidural analgesia, and patient-controlled intravenous analgesia.^[8,9]^ It is undeniable that these analgesic models have some shortcomings. More and more studies have shown that acupuncture assisted anesthesia (AAA) has become an important part of multimodal non opioid analgesia, which will help to improve the postoperative ERAS.^[9,10]^ AAA can effectively reduce the dosage of anesthetics during operation and postoperative analgesics, reduce the incidence of postoperative nausea and vomiting, reduce postoperative complications, shorten hospital stay, and so on.^[11]^ In recent years, transcutaneous electrical acupoint stimulation (TEAS), electroacupuncture, wrist ankle acupuncture, and other acupuncture related technologies combined with anesthesia have been applied to video assisted thoracic surgery, which has significant advantages in postoperative analgesia, protecting organ function and reducing inflammatory reaction.^[12–14]^ There is a study is evaluating the efficacy and safety of plum-blossom needle acupuncture combined with Tramadol in improving chronic postsurgical pain after lobectomy in non-small cell lung cancer patients.^[15]^ Our study does not limit the types of chest diseases in thoracoscopy, such as pneumonectomy, lobectomy, radical resection of lung cancer. Our study plan to evaluate the efficacy and safety of AAA for the treatment of postoperative pain after thoracoscopy based on randomized controlled trials (RCTs), to provide more evidence to support the management of postoperative pain.

## 2 Methods

### 2.1 Study registration

This systematic review protocol has been registered on the website of International Platform of Registered Systematic Review and Meta-analysis Protocols. The approved registration number is International Platform of Registered Systematic Review and Meta-analysis Protocols 2021120129.

### 2.2 Inclusion criteria for this study

#### 2.2.1 Types of studies

All RCTs with AAA for postoperative pain of thoracoscopy were included. Considering the language limitations of the study investigator, only relevant RCTs published in English or Chinese will be included. The non-randomized clinical trials, case reports, reviews, protocols, animal experimental researches, and ongoing or uncompleted trials will be excluded.

#### 2.2.2 Types of participants

The inclusion criteria of patient for this study:

(1) Patients who underwent thoracoscopy under AAA with no limitation of the types of chest diseases or will be included in our study.
(2) There are no restrictions on age, gender, race, education, socioeconomic status, types of disease, et al.

The exclusion criteria of patient for this study:

(1) The adjacent nerves of acupoints, such as median, ulnar and radial nerves, are confirmed to have impaired function and hypoesthesia due to cervical spondylosis or trauma.
(2) There is local infection or chronic inflammation on the surface of the acupoint stimulation site, or the electrode cannot be applied. Severe pulmonary infection, bronchitis, airway hyperresponsiveness, and large amount of expectoration are expected to have a great impact on postoperative extubation.
(3) Patients with severe central system diseases, rheumatic immune system diseases and severe mental diseases before operation.
(4) Patients who have participated in other clinical trials in recent 4 weeks.
(5) Bronchodilation, pulmonary tuberculosis, and severe pleural adhesion are not suitable for thoracoscopic surgery.
(6) Patients who cannot cooperate to complete the study plan, including those with language difficulties, infectious diseases, or other medical history.

#### 2.2.3 Type of interventions

The inclusion criteria of intervention in treatment group for this study:

(1) The treatment group underwent AAA.
(2) There is no limitation on the acupuncture measures in the treatment group, include acupuncture, electroacupuncture, TEAS, et al.
(3) The intervention time, frequency, and the electrical stimulation wave of acupuncture is not limited.
(4) There is no limitation on the method of anesthesia during surgery, we included both general anesthesia and other anesthesia methods, such as general anesthesia or epidural anesthesia.

The exclusion criteria of intervention in treatment group for this study:

(1) animal experiments and the treatment group without applying acupoints and meridians.

The inclusion criteria of intervention in control group for this study: the control group treated with sham-acupuncture, blank control, or the same intervention as the treatment group other than acupuncture will also be included. The exclusion criteria of intervention in control group for this study: when the control group underwent different frequency, electrical stimulation waveform, intervention time, and other forms of acupuncture compared with the treatment group, it will be excluded.

#### 2.2.4 Types of outcome measures

The primary outcomes consists of the indicator of the pain intensity, such as the visual analog scale, intraoperative anesthetic dosage, and the consumption of postoperative analgesics. As we know, the application dose of postoperative analgesics is positively correlated with the intensity of postoperative pain. Therefore, postoperative pain score and postoperative analgesic dose are important indexes to evaluate perioperative pain management.^[16]^ The secondary measures are Subjective Sleep Quality Score, the total sleep time after operation, residence time in the anesthesia recovery room, the duration of hospitalization, and the incidence of adverse reactions and serious events, such as nausea, vomiting, dizziness, chest tightness, cough, hypotension, hypertension, arrhythmia, et al. Lack of sleep can lead to increased sensitivity to pain. High levels of pain during the day and painkillers are important predictors of poor sleep the next night.^[17]^ Therefore, improving postoperative sleep quality can not only reduce postoperative pain, but also enhance the efficacy of postoperative analgesics; In addition, the improvement of postoperative pain is also conducive to improving postoperative sleep. Therefore, this study evaluated the postoperative sleep status as a secondary indicator related to postoperative pain.

### 2.3 Data sources

#### 2.3.1 Electronic searches

Our study will perform a comprehensive and systematic literature search mainly on 7 electronic databases from their inception up to November 30, 2021. The specific databases are as following: PubMed, the Cochrane Library, Embase, China National Knowledge Infrastructure, Chongqing VIP Information, and WanFang Data, Chinese Biomedical Database. Medical Subject Heading terms and free-text terms with logical operators will be adopted in the strategies of researches. Asterisks, regarded as truncation symbols, will play an important role in searching for all designs with asterisks. The retrieval strategy in PubMed is considered as an example in the researching process, which is presented in Table 1.

**Table 1:** Search strategy of PubMed.

Number	Search items
1	“acupuncture”[MeSH Terms]
2	“acupuncture therapy”[Title/Abstract]
3	“electroacupuncture”[Title/Abstract]
4	“ear acupuncture”[Title/Abstract]
5	“auricular acupuncture”[Title/Abstract]
6	“TEAS”[Title/Abstract]
7	“transcutaneous electrical acupoint stimulation”[Title/Abstract]
8	“acupuncture point”[Title/Abstract]
9	“acupoint”[Title/Abstract]
10	“acupressure”[Title/Abstract]
11	“moxibustion”[Title/Abstract])
12	1 or 2 or 3 or 4 or 5 or 6 or 7 or 8 or 9 or 10 or 11
13	“anesthesia”[MeSH Terms]
14	“narcosis”[Title/Abstract]
15	“narcotism”[Title/Abstract]
16	“anaesthetization”[Title/Abstract]
17	“analgesia”[Title/Abstract]
18	“general anesthesia”[Title/Abstract]
19	“nerve block”[Title/Abstract]
20	“patient controlled epidural analgesia”[Title/Abstract]
21	“patient controlled intravenous analgesia”[Title/Abstract]
22	“PCEA”[Title/Abstract]
23	“PCIA”[Title/Abstract]
24	13 or 14 or 15 or 16 or 17 or 18 or 19 or 20 or 21or 22 or 23
25	“thoracoscopy”[MeSH Terms]
26	“video assisted thoracoscopic surgery”[Title/Abstract]
27	“VATS”[Title/Abstract]
28	“thoracoscopic surgery”[Title/Abstract]
29	25 or 26 or 27 or 28
30	“Pain, Postoperative”[MeSH Terms]
31	“postoperative pain” [Title/Abstract]
32	“pain management ”[Title/Abstract]
33	“postoperative analgesi∗”[Title/Abstract]
34	“pain management”[Title/Abstract]
35	“ache∗”[Title/Abstract]
36	“suffering∗”[Title/Abstract]
37	“discomfort”[Title/Abstract]
38	30 or 31 or 32 or 33 or 34 or 35 or 36 or 37 or 38
39	12 and 24 and 29 and 38

MeSH = medical subject terms, PCEA = patient-controlled epidural analgesia, PCIA = patient-controlled intravenous analgesia, TEAS = transcutaneous electrical acupoint stimulation, VATS = video-assisted thoracoscopic surgery.

#### 2.3.2 Searching for other resources

The ongoing or unpublished studies relevant to our study will be searched from PROSPERO, ClinicalTrials.gov, Chinese Clinical Trial Registry, the US National Institutes of Health register, the World Health Organization International Clinical Trials Registry Platform. We will also conduct manual retrieval of relevant trials in books, journals, academic reference reporting, and the field of potential gray literature.

### 2.4 Data collection and analysis

#### 2.4.1 Selection of studies

**Figure 1:**
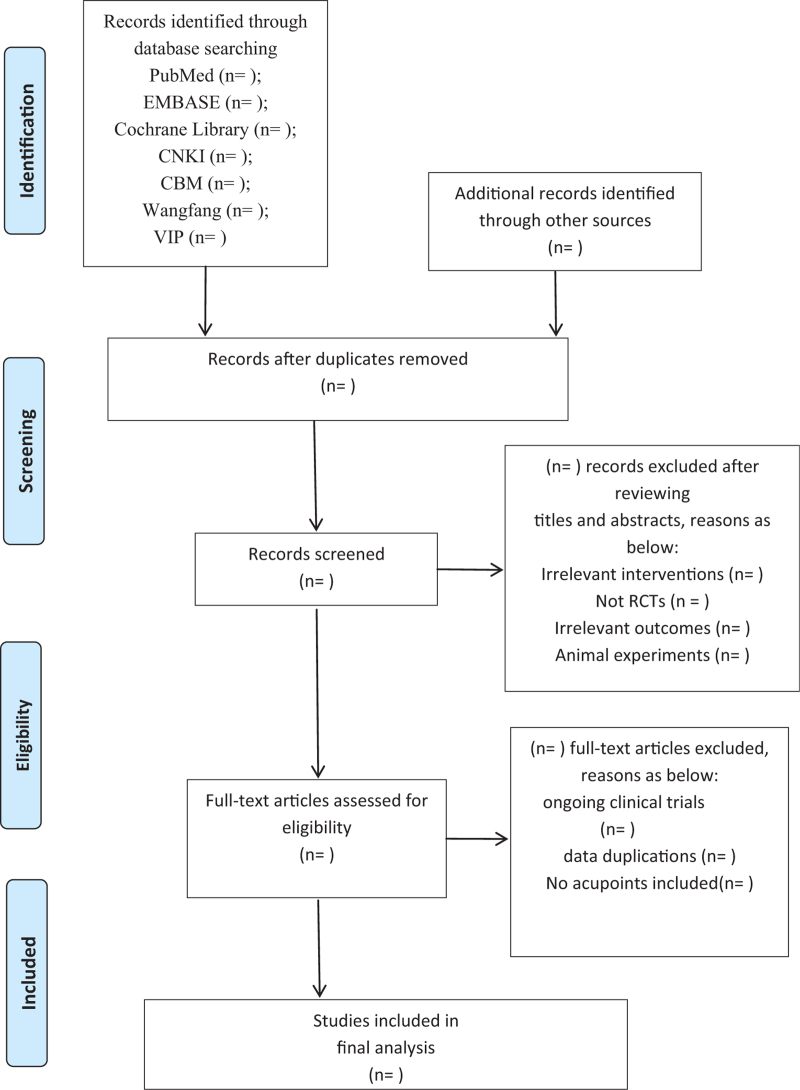
PRISMA flow diagram of the study process. PRISMA = preferred reporting items for systematic review and meta-analysis.

All the literature retrieved from the databases will be imported into the Document Explorer (EndNote X9 software) and then complete the selection process of repeated literature automatically. Two researchers (Liu and Meng) will read the titles and abstracts of the literature to eliminate the irrelevant, and then communicated with each other. Further full-texts will be downloaded and selected whether it is consistent with our included criteria. The disagreements in any of the above processes, the 2 researchers (Liu and Meng) will communicate with the third party (Zhao) for a final judgment. The flow chart of the selection procedure followed by Preferred Reporting Items for Systematic Reviews and Meta-Analysis Protocol (PRISMA-P) was shown in Figure 1.

#### 2.4.2 Data extraction

Two independent investigators will be responsible for extracting and tabulating the data regarding the scheduled program. Discrepancies will be resolved via communicating and deciding with the third party (Yan). The necessary e-mails or telephones with the correspondent authors will be conducted when the data is incomplete or ambiguous. In the multi-arm RCTs, our study will only extract the data related to our criteria according to the specific interventions.

The predesigned data extraction items: first author name, publication year, types of thoracic surgery, such as pneumonectomy, lobectomy, radical resection of lung cancer, sample sizes, interventions, anesthesia mode, intervention measures and time, acupoints, waveform, electrical stimulation frequency, outcome measures, and adverse events.

#### 2.4.3 Dealing with missing data

We will resort to the corresponding author of the literature via e-mail or telephone for the missing or inexplicit data. If no response is received from the author or the data is confirmed to have been lost, we will exclude the relevant literature. The process of finding data will be described in detail in the discussion section.

#### 2.4.4 Assessment of risk of bias in included studies

Our study will assess of risk of bias in included studies following the Cochrane risk of bias tool,^[18]^ which is consisted of random sequence generation, allocation concealment, blinding of participants and personnel, blinding of outcome assessment, selective reporting, incomplete outcome data, and other risks of bias, in which the “other risks of bias” are included as following:

(1) Whether the reported baseline between the treatment group and the control group are comparable;
(2) Whether the criteria for inclusion or exclusion are clear;
(3) Whether the experimental design is clinical or feasible;
(4) Whether there is a crucial conflict of interest that leads to increased bias.

The level of the assessment can be divided to 3 parts called “low-risk”, “high-risk”, or “unclear-risk”. Disagreements will be resolved via communications between the 2 investigators or after discussion with a third party (Yan and Wang).

#### 2.4.5 Statistical analysis

Statistical analysis of the included RCTs will be performed with the Stata 15.0 software. The mean difference or standard mean difference with 95% confidence interval was used for continuous variables. The relative risk with 95% confidence intervals was utilized for dichotomous variables. In terms of the multi-arm RCTs that met the included criteria, meta-analysis will merge the data into a unified intervention group prudently. The magnitude of heterogeneity will be measured using the heterogeneity *I*^*2*^ statistic indicator. Different data processing models will be used according to different data results: a fixed-effects model will be used for pooled data when *I*^*2*^ < 50%; a random-effects model will be used when *I*^*2*^ ≥ 50%. The chi-squared statistic will be performed to detect a heterogeneity test for each merged analysis. If *I*^*2*^ ≥ 50%, the synthesized studies will be considered as an indicator of high heterogeneity. When significant heterogeneity exists in the results of the combined data, sensitivity analysis will be used to assess the stability of the results of each meta-analysis and help identify the sources of heterogeneity by screening the literature with a higher risk of bias. Descriptive analysis will be conducted when meta-analysis is not appropriate.

#### 2.4.6 Subgroup analysis

Subgroup analysis will be performed to identify the possible factors that contributed to the heterogeneity. The influencing factors can be divided into the following categories:

(1) Different acupuncture related technology, such as acupuncture, electroacupuncture, TEAS;
(2) Different types of anesthesia during surgery, such as general anesthesia or epidural anesthesia;
(3) Different intervention duration;
(4) Different intervention time, such as preoperative time, intraoperative time, or postoperative time;
(5) Different types of thoracic surgery, such as pneumonectomy, lobectomy, radical resection of lung cancer;
(6) Different waveforms or frequencies of electrical stimulation.

#### 2.4.7 Assessment of reporting biases

Funnel plots will be performed to evaluate the publication bias if there are enough amounts of RCTs (n ≥ 10) in each of the meta-analyses. An Egger regression test will be used to quantitatively evaluate funnel plot asymmetry.^[19]^

### 2.5 Paper writing

The protocol of our study will follow the PRISMA-P statement guideline.^[20]^ Besides, our study will be performed in compliance with the PRISMA statement guidelines.^[21]^

### 2.6 Quality of the evidence

The Grading of Recommendations Assessment, Development, and Evaluation approach will be conducted to evaluate the quality of evidence for outcomes.^[22]^ And the level of evidence will be divided into 4 categories: very low, low, moderate, and high.

## 3 Discussion

As far as we know, with the promotion of the concept of “minimally invasive” surgery, thoracoscopy is a minimally invasive surgery in thoracic surgery. However, it is undeniable that the postoperative pain caused by it is a key problem that needs urgent attention and solution.^[23,24]^ Acupuncture combined anesthesia is often used in clinic because of its safety, less adverse reactions, less physiological interference, and rapid postoperative recovery. Adequate and effective analgesia after thoracic surgery is an important part of ERAS. At present, studies have shown that the application of acupuncture combined anesthesia in thoracoscopy has achieved good curative effect. However, there is no relevant systematic evaluation. The addition of general anesthetics on the basis of acupuncture continuous stimulation treatment can make up for the large individual differences in analgesia of simple acupuncture anesthesia technology, the insufficient analgesic effect to eliminate surgical stimulation, and basically no muscle relaxation required for surgery.^[25]^ Our study is the first meta-analysis of the effectiveness and safety of acupuncture combined anesthesia in pain management after thoracoscopy, in order to provide strong evidence for clinical support.

## 4 Ethics and dissemination

Ethics approval is not required in our systematic review and meta-analysis which is based on published randomized clinical trials.

## Author contributions

**Conceptualization:** Xinyi Liu, Dan Meng.

**Data curation:** Dan Meng, Chunchun Yan.

**Formal analysis:** Yan Chunchun.

**Investigation:** Xinyi Liu, Dan Meng, Qinyu Zhao.

**Methodology:** Xinyi Liu, Qinyu Zhao, Jingyu Wang.

**Supervision:** Xinyi Liu, Dan Meng, Chunchun Yan.

**Validation:** Dan Meng, Qinyu Zhao, Jingyu Wang.

**Visualization:** Dan Meng, Jingyu Wang.

**Writing – original draft:** Xinyi Liu, Dan Meng, Qinyu Zhao.

**Writing – review & editing:** Xinyi Liu, Jingyu Wang.
